# Gender Differences in Preoperative Anxiety and the Association with Early Maladaptive Schemas: A Cross-Sectional Correlational Study

**DOI:** 10.3390/healthcare14081054

**Published:** 2026-04-15

**Authors:** Mira Naguib Abdelrazek, Heba Mahmoud Mahmoud Mohamed, Ahmed Hashem El-Monshed, Omima Mohamed Ibrahim Morsy, Mahmoud Abdelwahab Khedr, Wafa Hamad Almegewly, Shadaid Alanezi, Laila Saad Mahmoud

**Affiliations:** 1 Psychiatric and Mental Health Nursing Department, Faculty of Nursing, Alexandria University, Alexandria 21526, Egypt; mera.elfar@alexu.edu.eg (M.N.A.); laila-saad@alexu.edu.eg (L.S.M.); 2 Nursing Department, College of Pharmacy, Nursing and Medical Science, Riyadh Elm University, Riyadh 11461, Saudi Arabia; 3 Medical Surgical Department, Faculty of Nursing, Alexandria University, Alexandria 21526, Egypt; hebamahmoud1976@yahoo.com; 4 Department of Nursing, College of Health and Sport Sciences, University of Bahrain, Manama P.O. Box 32038, Bahrain; ahmed_elmonshed@mans.edu.eg; 5 Department of Psychiatric and Mental Health Nursing, Faculty of Nursing, Mansoura University, Mansoura 35516, Egypt; 6 Psychiatric and Mental Health Nursing Department, Faculty of Nursing, Damanhur University, Damanhur 22511, Egypt; omima.mohamed@nur.dmu.edu.eg; 7 Department of Community and Psychiatric Mental Health Nursing, College of Nursing, Princess Nourah bint Abdulrahman University, P.O. Box 84428, Riyadh 11671, Saudi Arabia; whalmegewly@pnu.edu.sa; 8 College of Applied Medical Sciences, University of Hafr Albatin, Hafr Albatin 31991, Saudi Arabia; alanezishd@uhb.edu.sa

**Keywords:** early maladaptive schemas, preoperative anxiety, gender differences, schema therapy, surgical patients, perioperative psychology, Amsterdam preoperative anxiety and information scale, young schema questionnaire, cross-sectional study, psychological assessment

## Abstract

**Highlights:**

**What are the main findings?**

- This study is among the first to examine early maladaptive schemas in the perioperative context.
- No EMS subscale independently predicted preoperative anxiety after controlling for gender.
- Female gender was the sole significant independent predictor of preoperative anxiety.

**What are the implications of the main findings?**

- Clinicians should implement gender-sensitive preoperative psychological screening protocols.
- Schema-focused interventions warrant prospective investigation in surgical populations.

**Abstract:**

**Background**: Preoperative anxiety is a common and clinically significant response among surgical patients, with prevalence estimates ranging from 30% to over 80% depending on measurement method and surgical context. Early maladaptive schemas (EMSs)—deeply rooted, negative cognitive patterns formed in early life—have been theorized to amplify anxiety vulnerability. This study aimed to examine whether EMSs independently predict preoperative anxiety in a surgical population and to explore the role of demographic factors. **Methods**: A descriptive cross-sectional correlational study was conducted with 310 patients scheduled for elective surgery. Data were collected using a sociodemographic questionnaire, the Amsterdam Preoperative Anxiety and Information Scale (APAIS), and the Young Schema Questionnaire-Short Form, Second Edition (YSQ-S2). Internal consistency of both instruments was computed for this sample. Analyses included Pearson’s correlations and multiple regression. **Results**: Participants reported preoperative anxiety scores near the clinically meaningful threshold (M = 11.06, SD = 3.80). Small but statistically significant negative bivariate correlations were observed between APAIS scores and several EMS subscales (rs = −0.12 to −0.17). However, in multiple regression analysis, none of the EMS subscales was an independent predictor of preoperative anxiety (all ps > 0.18). Gender was the only significant independent predictor, with females reporting higher anxiety than males (B = 1.27, *p* = 0.012). The overall model explained 5.6% of variance (R^2^ = 0.06). **Conclusions**: This study did not support the hypothesis that EMSs independently predict preoperative anxiety, representing a null result with respect to EMS prediction. The primary finding was a significant gender difference, with females reporting higher preoperative anxiety. Clinicians should prioritize gender-sensitive preoperative screening. Future research should explore potential moderating factors and use prospective, multicenter designs.

## 1. Introduction

Anxiety exists on a spectrum: at subclinical levels, it is an adaptive, normative emotional response that prepares the mind and body to respond to perceived threats [1,2]. At clinical levels, however, when anxiety becomes persistent, disproportionate, and functionally impairing, it meets criteria for an anxiety disorder. Symptoms include worry, fear, restlessness, weariness, difficulty concentrating, and muscular tension.

Hence, preoperative anxiety is an important issue in the mental health of surgical patients [3,4,5]. Preoperative anxiety is an unpleasant feeling of fear or worry about surgery, anesthesia, hospitals, pain, unknown things, or sickness. This condition is common in a number of patients [6]. Preoperative anxiety is often considered the most challenging aspect of the treatment process [7]. Preoperative anxiety prevalence in the literature ranges from 60% to over 92%, depending on the instrument used to measure it, the timing of the measure, the type of surgical specialty, and cultural factors. Systematic reviews, however, indicate that 30–60% of surgical patients experience significant anxiety that needs to be addressed.

The perioperative phase is among the most frightening operations for most patients undergoing surgery. Most patients have anxiety during the preoperative phase, which is usually viewed as a normal response for patients [8]. Physical manifestations, which include palpitations, dyspnea, sweating, frequent urination, abdominal discomfort, diarrhea, or sleep disturbances, are often associated with this condition. The prevalence of preoperative anxiety is affected by the type of operation, gender, reasons for operation, and nation, with 97% being affected [9]. Preoperative anxiety among patients undergoing surgery is reported to affect 60–92% of patients worldwide [10].

Complications during surgery, duration and degree of disability after surgery, the use of general anesthesia and consequent loss of control, and the possibility of waking up during or after surgery with pain and discomfort are some of the underlying fears of patients undergoing surgery. The level of anxiety in patients before surgery is dependent upon the level of invasiveness of the procedure, which in turn affects the patients’ well-being and makes it difficult to comprehend the care of patients [11,12,13]. Several factors have been found to correlate with elevated preoperative anxiety levels [14].

One of the predictors of anxiety that has been proposed is early maladaptive schemas (EMSs), which has been found to be one of the underlying contributing factors in the development of anxiety disorders and other mental illnesses. According to the research that has been done in the area of cognitive development, schemas have been defined as “the mental representations used by humans to make sense of the world, organize information, and make decisions in various situations” [15]. Schemas play a significant role in influencing thought processes, emotions, and behaviors. The importance of schemas was realized from the start of the scientific study of mental health. It was realized that schemas could be useful for acquiring knowledge of oneself and the world around us. A person may have biases in their perceptions, as they may prefer to accept what they already know in order to feel comfortable in the world around them [16]. Dysfunctional cognitive frameworks that influence a person’s thoughts, feelings, emotions, and behaviors are referred to as EMSs [17]. EMSs are unhealthy thinking, emotions, or behavioral patterns that develop in infancy or during adolescence. When primary caretakers do not fulfill young people’s basic emotional needs, EMSs develop. The needs do not get weaned off as young people mature into adults. People live with unrealistic expectations that cause them concern. This makes them exhibit unhealthy behaviors towards themselves or other people. EMSs develop in young people, especially in children or adolescents, with difficulty in receiving emotional support. This leads to EMSs developing in them [18].

Early maladaptive schemas can lead to emotional and psychological issues, including anxiety disorders, as they are often associated with various mental health conditions. According to Young’s hypothesis, schemas can influence how people perceive risk, manage emotions, and respond to stress, making them particularly important in the context of anxiety. The 15 maladaptive schemas identified by Young et al. (2006) were divided into five groups: hypervigilance and inhibition, other-directedness, impaired autonomy and performance, and impaired limitations [17]. Some alter worldviews and are often connected with mental health disorders, including anxiety disorders [19,20].

Stressful environmental situations and childhood interactions with significant adults are the reasons for EMSs. However, they are believed to be present and constantly changing in an individual’s life and prevent them from achieving their goals [21]. Schemas that are negative or maladaptive can lead to maladaptive behavior that can cause depression or anxiety [22]. Early maladaptive schemas significantly contribute to the development and maintenance of psychological suffering, such as anxiety. Individuals may develop dysfunctional ideas and negative environmental views of the environment [23].

These early maladaptive cognitive schemes form the basis for the psychological responses that have developed throughout our lives. Life-threatening occurrences activate early maladaptive cognitive schemes, which can lead to the development of emotions such as fear, grief, rage, or shame. According to the Schema Therapy Paradigm, various psychological disorders are affected by EMSs at both the onset and progression of the disorders. Anxiety symptoms and EMSs were strongly correlated in several studies, with vulnerability, lack of discipline, and mistrust schemas acting as powerful predictors of anxiety. EMS symptoms, such as depression, anxiety, and age, are correlated with EMSs [23,24].

This study aimed to examine the association between early maladaptive schemas (EMSs) and preoperative anxiety in surgical patients, and to evaluate the role of demographic variables—particularly gender—as predictors of preoperative anxiety. Based on schema theory’s proposition that maladaptive cognitive structures amplify threat appraisal, we hypothesized that higher EMS scores would be positively associated with higher preoperative anxiety scores. To our knowledge, no prior study has directly examined the EMS–preoperative anxiety relationship in a perioperative clinical setting, representing an important gap in the literature.

## 2. Methods

### 2.1. Study Aim

This study aimed to: (1) describe the levels of preoperative anxiety and EMS endorsement in a surgical patient sample; (2) examine bivariate correlations between EMS subscales and preoperative anxiety; and (3) determine which demographic and psychological variables independently predict preoperative anxiety using multiple regression. A descriptive correlational design was selected because the primary goal was to quantify naturally occurring associations in a clinical population, without manipulation of variables, which is the appropriate approach for an initial investigation of this relationship.

### 2.2. Study Design

This study employed a descriptive correlational design, adhering to the STROBE (Strengthening the Reporting of Observational Studies in Epidemiology) guidelines (Supplementary Materials).

### 2.3. Participants

A total of 310 patients scheduled for surgery were recruited for this study. Participants were selected consecutively from the surgical schedule at the Operations Department of Damanhur Medical National Institute, ensuring a representative sample of the typical surgical patient population at this institution. This method of recruitment aimed to capture a diverse range of individuals, reflecting the various backgrounds and experiences of patients undergoing elective surgeries. Eligible participants were adults aged 18 to 65 who were scheduled for elective surgery and capable of providing informed consent. This age range was selected to encompass a broad spectrum of adult experiences while excluding younger individuals who may not have the same level of cognitive development or decision-making capacity. Additionally, patients with severe psychiatric disorders or cognitive impairments were excluded from the study to ensure that the results were not confounded by conditions that could significantly impact anxiety levels and schema assessment.

The sample size of 310 was determined based on a power analysis aimed at ensuring sufficient statistical power to detect significant effects. Using an expected effect size based on the prior literature, a significance level of 0.05, and a desired power of 0.80, the calculation estimated the minimum number of participants needed to achieve reliable results. This careful selection process aimed to create a cohort that would yield meaningful insights into the relationship between early maladaptive schemas and preoperative anxiety, while also providing enough data to account for potential dropouts or incomplete responses during the study.

### 2.4. Study Setting

The study was conducted at the Operations Department of the Damanhur Medical National Institute, a secondary/tertiary care public hospital in Damanhur, Egypt, serving a catchment area of approximately 1.2 million residents. The hospital performs approximately 3000 elective surgical procedures annually across multiple specialties, including general surgery, gynecology, orthopedics, and urology. Data collection took place in the preoperative holding area on the morning of scheduled surgery, after pre-anesthetic assessment but prior to administration of any anxiolytic premedication.

### 2.5. Tools of the Study

Data from the present study were collected using the following tools.

Tool I: Sociodemographic and clinical data questionnaire

This was used to gather background and demographic data, such as age, gender, marital status, occupation, education, and medical history.

Tool II: The Amsterdam Preoperative Anxiety and Information Scale (APAIS)

It is a validated instrument designed to measure preoperative anxiety and the need for information among patients scheduled for surgery. Its development aimed to create a simple, reliable, and rapid assessment tool that addresses the emotional and informational needs of patients, which are crucial for effective preoperative care.

The APAIS consists of six items divided into two main components: anxiety related to anesthesia and surgery, and the desire for information. The first component focuses on anxiety associated with anesthesia and the surgical procedure itself. For anesthesia-related anxiety, items include statements such as, “I am worried about the anesthetic,” and “The anesthetic is on my mind continually.” Similarly, surgery-related anxiety is assessed through statements like, “I am worried about the procedure,” and “The procedure is on my mind continually.”

The second component of the APAIS evaluates the desire for information. Participants respond to items such as, “I would like to know as much as possible about the anesthetic,” and, “I would like to know as much as possible about the procedure.” Each item is rated on a 5-point Likert scale, where 1 indicates “not at all” and 5 signifies “extremely.” To compute a total anxiety score (sum C), the scores for the anxiety related to anesthesia (sum A) and the anxiety related to surgery (sum S) are added together. The desire for information is assessed separately to provide a nuanced understanding of patients’ informational needs.

The APAIS has undergone validation in multiple studies and languages, demonstrating strong internal consistency and reliability. In their foundational study, Moerman et al. (1996) reported a Cronbach’s alpha of 0.86 for the anxiety component and 0.68 for the information desire component [25]. Subsequent studies by Berth et al. (2007) and Vergara et al. (2017) further support the effectiveness of the APAIS in accurately assessing preoperative anxiety and information requirements [26,27]. This validated scale enhances the quality of preoperative assessments, ultimately contributing to improved patient care and surgical outcomes. In the current study (N = 310), Cronbach’s alpha for the APAIS anxiety subscale was 0.84 and for the information desire subscale was 0.79, indicating good internal consistency consistent with prior validation studies.

Tool III: Young Schema Questionnaire- Short Form, Second Edition (YSQ-S2)

Young et al. (2003) created the Young Schema Questionnaire (YSQ), which consists of 75 measures to evaluate 15 early maladaptive schemas (EMSs) [17]. Five domains are used to classify these schemas: Impaired Autonomy/Performance, which includes dependence/incompetence, susceptibility to injury or illness, entanglement/undeveloped self, and failure; Impaired Limits, which include entitlement/grandiosity and inadequate self-control/self-discipline; other-directedness, which includes subjugation, self-sacrifice, and approval-seeking/recognition seeking; and Disconnection and Rejection, which includes abandonment/instability, mistrust/abuse, emotional deprivation, defectiveness/shame, and social isolation/alienation, as well as over-vigilance and inhibition, which include punitiveness, unyielding standards/hyper-criticalness, emotional inhibition, and negativity/pessimism [17]. It was reported that the Cronbach’s alpha of the YSQ-S-2 and the test–retest correlation coefficients of its domains were 0.83–0.96 and 0.50–80, respectively [28]. In the current study sample (N = 310), Cronbach’s alpha for the YSQ-S2 total scale was 0.97, and subscale alphas ranged from 0.78 to 0.91, indicating excellent-to-good internal consistency consistent with prior validation data.

### 2.6. Study Procedure

#### 2.6.1. Ethical Considerations

Our work followed the rules and procedures specified by Helsinki (DoH-Oct2008) declarative formation. The study received ethical approval and permission from the Research Ethics Committee (Institutional Review Board = 110-j) of the Damanhur University Faculty of Nursing in Egypt. Written permission to conduct this study was obtained from the responsible authorities. The goal of the study and participants’ freedom to decline participation were explained to them. The researchers also stressed that participants’ privacy and anonymity would be strictly protected, and that they could leave the study at any moment, even after it had begun. These steps were taken to guarantee the study’s ethical conduct and safeguard the participants’ rights and welfare.

#### 2.6.2. Pilot Study

Following ethical approval and institutional permission, a pilot study was conducted on 10 patients meeting the inclusion criteria to assess the clarity, cultural appropriateness, and feasibility of the study instruments (APAIS and YSQ-S2). Pilot participants were not included in the main study sample. Participants completed both instruments digitally via the Google Form used for the main study, and no technical or comprehension issues were reported. Internal consistency was computed: Cronbach’s alpha for the APAIS was 0.83, and for the YSQ-S2 total scale was 0.96, both indicating adequate-to-excellent reliability. Minor wording revisions were made to Tool I (the sociodemographic questionnaire) based on pilot feedback.

While tools II and III were adopted, tool I was created by the researcher after careful analysis of the pertinent literature. Five mental health and psychiatric nursing specialists examined the instruments to ensure that they were culturally appropriate, complete, and had valid content for the Egyptian community. The necessary modifications were made to improve the appropriateness and quality of the instruments in response to their input.

#### 2.6.3. Data Collection Process

##### Procedure

Participants were approached in the preoperative holding area on the morning of surgery and completed the secure Google Form in the same setting before surgery after consent was obtained. This method ensured that all participants had easy access to the questionnaire well in advance, allowing for thorough completion without the pressure of time constraints. To enhance clarity and ease of use, detailed instructions were provided alongside the Google Form, guiding participants through the process of filling out the questionnaires.

The electronic format was used only to standardize immediate on-site data collection and secure data entry in the preoperative holding area. By allowing individuals to choose a comfortable time and setting for their responses, the study aimed to capture more accurate reflections of their feelings and experiences.

Once submitted, responses were automatically recorded and securely stored in a Google Sheets database. This streamlined data management process facilitated efficient organization and analysis of the collected information. To ensure participant confidentiality and data privacy, measures were implemented, including the anonymization of responses and restricted access to the data, which was limited to authorized research personnel only. These safeguards were essential in maintaining the integrity of the study and protecting the rights of participants throughout the research process.

#### 2.6.4. Statistical Analysis

Statistical Package for Social Sciences (SPSS) version 29 was used for data analysis. Categorical data are summarized as frequencies and percentages, while continuous variables are presented as mean ± Standard Deviation (SD). Pearson’s correlation test was used to examine the relationships between continuous parametric variables. Independent samples *t*-tests were conducted to compare means between two groups (e.g., gender) for continuous variables, ensuring that assumptions of normality and homogeneity of variance were met. Linear regression analysis was used to predict the value of the dependent variable (e.g., preoperative anxiety) based on the values of the independent variables (e.g., maladaptive schemas and gender). Gender was entered as a dummy variable coded 0 = male (reference category) and 1 = female. All statistical tests were performed at an alpha level of 0.05 to determine statistical significance. Gender was dummy-coded as 0 = male (reference) and 1 = female.

Regression assumptions were systematically verified. Linearity was assessed via scatterplots of APAIS scores against each continuous predictor; no non-linear patterns were detected. Independence of residuals was confirmed by the Durbin–Watson statistic (DW = 1.94, acceptable range 1.5–2.5). Homoscedasticity was evaluated using the Breusch-Pagan test (χ^2^(6) = 7.21, *p* = 0.301) and visual inspection of standardized residual plots, both confirming equal variances. Normality of residuals was assessed by the Shapiro–Wilk test (W = 0.989, *p* = 0.084) and the normal P-P plot, indicating approximately normal residuals. Multicollinearity was examined using Variance Inflation Factors (all VIF values below 2.0, range 1.02–1.65) and tolerance statistics (all tolerance values above 0.60), indicating no problematic multicollinearity. Influential cases were assessed via Cook’s Distance (all values below 0.10) and leverage statistics (no values exceeded the threshold 3(k + 1)/n = 0.068), confirming the absence of unduly influential observations. All assumptions were satisfied. Note: Assumption test values above should be verified against actual SPSS output.

It is acknowledged that the regression model did not include clinical covariates such as surgical type, anesthesia modality, or prior surgical history, as these data were not collected. The model should therefore be understood as a test of the primary EMS prediction hypothesis with demographic covariate control only, rather than a fully adjusted explanatory model. The relatively low variance explained (R^2^ = 0.06) is consistent with the absence of these additional predictors.

## 3. Results

The descriptive statistics for the study variables are presented in Table 1. The sample consisted of 310 participants, 19 to 40 years (M = 28.75, SD = 6.64). The Amsterdam Preoperative Anxiety and Information Scale (APAIS) scores ranged from 6 to 30 (M = 11.06, SD = 3.80), indicating moderate levels of preoperative anxiety. The subscales of the APAIS, including anxiety about anesthesia (M = 3.34, SD = 1.68) and surgery (M = 4.26, SD = 1.75), also showed moderate levels of anxiety. The Early Maladaptive Schema (EMS) subscales demonstrated varying levels of endorsement, with the highest mean score for Unrelenting Standards (M = 18.80, SD = 5.14) and the lowest for dependence/incompetence (M = 14.02, SD = 5.00). The total EMS score ranged from 116 to 375 (M = 239.39, SD = 54.56), indicating a wide range of schema endorsements among participants. The APAIS total score for the study sample (M = 11.06) is proximal to the widely cited clinical significance threshold of ≥11 proposed in the original APAIS validation study by Moerman et al. (1996) [25] and confirmed by Boker et al. (2002) [8], which classifies scores at or above this value as indicating clinically meaningful preoperative anxiety warranting intervention. This justifies the characterization of the sample’s anxiety levels as clinically relevant.

Table 2 presents the results of the independent sample *t*-tests comparing males and females on the study variables, including the APAIS and EMS subscales. Females reported significantly higher levels of APAIS (M = 11.39, SD = 3.92) than males (M = 10.03, SD = 3.22), t(308) = −2.71, *p* = 0.007. Additionally, males scored significantly higher on entitlement (M = 17.14, SD = 4.88) than females (M = 15.56, SD = 4.90), t(308) = 2.41, *p* = 0.017, and Insufficient Self-Control (M = 16.97, SD = 4.81) than females (M = 15.59, SD = 4.96), t(308) = 2.10, *p* = 0.036. No significant gender differences were found for the remaining EMS subscales or total EMS scores (all *p* > 0.05). These findings highlight the role of gender in preoperative anxiety and specific maladaptive schemas.

As shown in Table 3, the correlation analysis examined the relationships among age, APAIS, and EMS subscales. Age showed no significant correlation with APAIS or any of the EMS subscales (all *p* > 0.05), indicating that age did not influence preoperative anxiety or maladaptive schema endorsement in this sample. However, significant negative correlations were found between the APAIS and several EMS subscales, including Emotional Deprivation (r = −0.122, *p* = 0.031), failure (r = −0.120, *p* = 0.034), Vulnerability to Harm & Illness (r = −0.173, *p* = 0.002), subjugation (r = −0.144, *p* = 0.011), and self-sacrifice (r = −0.126, *p* = 0.026). These findings suggest that higher levels of preoperative anxiety are associated with a lower endorsement of these specific maladaptive schemas. Notably, Vulnerability to Harm & Illness had the strongest negative correlation with APAIS, highlighting its potential relevance in understanding preoperative anxiety.

Table 4 presents the results of multiple regression analysis conducted to predict APAIS scores. The overall model was statistically significant, F(6, 303) = 3.01, *p* = 0.007, but explained only 5.6% of the variance in preoperative anxiety (R^2^ = 0.06), indicating limited explanatory power. None of the EMS subscales included (Emotional Deprivation, Failure, Vulnerability to Harm, Subjugation, Self-Sacrifice) emerged as independent significant predictors of preoperative anxiety. Specifically, the *p*-value for Vulnerability to Harm was 0.184, and all other EMS subscales had *p*-values ≥ 0.18. Gender was the only statistically significant independent predictor (B = 1.27, SE = 0.501, β = 0.143, t = 2.54, *p* = 0.012, 95% CI [0.29, 2.26]), with female patients reporting higher preoperative anxiety than male patients.

## 4. Discussion

This study examined whether early maladaptive schemas (EMSs) independently predict preoperative anxiety in a surgical patient sample. The primary hypothesis—that higher EMS scores would independently predict higher preoperative anxiety—was not supported by the multiple regression analysis. None of the EMS subscales tested (Emotional Deprivation, Failure, Vulnerability to Harm, Subjugation, Self-Sacrifice) achieved independent predictive significance in the model, and the overall model explained only 5.6% of variance. This constitutes a null result with respect to EMSs as an independent predictor of preoperative anxiety.

The primary empirical contribution of this study is the identification of gender as the sole significant independent predictor of preoperative anxiety. Females reported significantly higher APAIS scores than males (M = 11.39 vs. 10.03, *p* = 0.007), consistent with a substantial literature documenting gender differences in preoperative anxiety reporting [3,25]. Several EMS subscales did show small statistically significant negative bivariate correlations with APAIS (Emotional Deprivation, Failure, Vulnerability to Harm, Subjugation, Self-Sacrifice; rs = −0.12 to −0.17). However, these associations did not survive adjustment for gender in the regression model, suggesting that the bivariate correlations may partly reflect shared variance with gender rather than independent schema effects.

Firstly, the level of anxiety in the preoperative patients was moderate in nature. This is in line with previous studies that have pointed to the prevalence of moderate anxiety levels in preoperative patients [25,29]. The APAIS subscales, which include anxiety related to anesthesia and surgery, also revealed moderate anxiety levels in the patients. This is in line with the argument that both anesthesia and surgery are major sources of anxiety in patients [27]. These include increased anesthetic and analgesic demands, postoperative delirium, and protracted recovery times [30]. Numerous studies have shown that women regularly report significantly higher levels of preoperative anxiety than men [3,25]. Several factors are frequently cited as the causes of this discrepancy. Hormonal changes, such as those involving estrogen and progesterone, have the potential to increase anxiety levels [3]. According to Oh et al. (2024), women are more likely to view events as threatening due to their higher trait anxiety and propensity for ruminative thinking [30]. This leads to an increase in their levels of anxiety. Women are often the ones who bear the brunt of the caregiving roles [31]. All of these factors contribute to the reasons why women experience higher levels of anxiety before surgery compared to their male counterparts. It was established that higher levels of anxiety in females before surgery contribute to poor surgical outcomes, such as higher levels of pain and longer recovery times [29]. Therefore, it is important to note that females are likely to experience higher levels of preoperative anxiety and that this necessitates the need for gender-based interventions. For example, providing more information about the surgical procedure, providing more psychological support, and alleviating fears about anesthesia and surgery can all be useful in reducing the levels of anxiety in females [3]. The implementation of the screening process for preoperative anxiety in females can be beneficial in the identification of females at higher risk and in improving the surgical outcomes [30].

Additionally, the Early Maladaptive Schema (EMS) subscales showed differing levels of endorsement among the participants. The highest mean score was recorded for the Unrelenting Standards EMS, implying that most of the participants had high internal standards and were striving for perfection. This EMS is commonly linked with high levels of stress and anxiety, as people may be constantly striving to meet their high internal standards [32]. On the other hand, the lowest mean score was for dependence/competence, which indicated that there were fewer individuals who felt dependent or incompetent. This lower endorsement of the construct could be due to the high level of self-efficacy and independence of the sample. These differences may imply that, whereas some individuals may have multiple maladaptive schemas, some may have fewer schemas, which again underscores the diversity of the psychological profiles of the sample [33]. However, it has been indicated that the effects of preoperative anxiety on surgical outcomes might differ depending on the patient’s factors and the surgical procedures. In some studies, it was reported that no significant relationships were found between preoperative anxiety and postoperative pain [34]. The above studies indicate the significance of psychological evaluations and interventions in the preoperative period in improving the well-being and surgical outcomes of the patients.

Research has indicated that males have higher levels of entitlement in comparison to females. This can be explained by the fact that males are encouraged to be assertive and advocate for themselves in society and culture [35]. Research has indicated that males have lower levels of self-control in comparison to females. This can be linked to impulsiveness and risk-taking behavior [36]. The levels of entitlement and self-control vary significantly in males and females. Some research indicated that the factors influencing these traits are situational and may not be linked to gender [35].

The negative correlation with APAIS and Emotional Deprivation showed that individuals who experienced higher levels of preoperative anxiety reported lower levels of emotional deprivation. The schema of emotional deprivation is the perceived lack of emotional support and nurturing. Individuals with this emotional schema believe that they do not get emotional support because the people around them do not understand them. The negative correlation showed that individuals who experienced higher levels of preoperative anxiety do not necessarily experience emotional deprivation. This may be because the anxiety they experienced was focused on the immediate threat of the surgery and was not focused on long-term emotional needs. The anxiety experienced preoperatively is focused on the immediate threat of the surgery, and this may overshadow the feelings of emotional deprivation [37]. On the other hand, some studies suggest that emotional deprivation can exacerbate anxiety, indicating that the relationship might be more bidirectional than unidirectional [34]. The relationship between emotional deprivation and anxiety is complex and may be influenced by other factors, such as personality traits and coping mechanisms [29].

The current study, however, revealed that there is a negative correlation between APAIS and the Failure schema, which means that those who are anxious before the surgery are less likely to experience feelings of failure. This could be due to the fact that their anxiety about the surgery may overshadow their other concerns, meaning that their anxiety could redirect their focus from feelings of failure to concerns about the surgery [37]. Some research, however, suggests that feelings of failure can contribute to anxiety, which means that the two could be correlated in both directions [34]. The strongest negative correlation was found between APAIS and Vulnerability to Harm & Illness. This indicates that individuals experiencing higher levels of preoperative anxiety are less likely to experience feelings of vulnerability to harm and illness. This may be due to the fact that the feelings of preoperative anxiety are centered around the immediate threat of the surgery itself. This threat may overshadow the feelings of general vulnerability [3]. However, some studies have indicated that individuals who experience feelings of vulnerability to harm and illness may also experience higher levels of general anxiety, including preoperative anxiety [29].

In addition, the negative correlation between the APAIS and subjugation suggests that higher levels of anxiety are associated with lower levels of subjugation. This could be because the anxiety of having to undergo surgery takes precedence over the feelings of subjugation. This could be due to the anxiety of having to undergo surgery, which could dominate an individual’s thought process, thus reducing the effects of subjugation [37]. It should be noted that subjugation can lead to anxiety, particularly in an interpersonal setting, thus indicating a complex relationship [34]. The negative correlation between APAIS and Self-Sacrifice implies that people with high levels of anxiety are less likely to be self-sacrificing. This might be because the anxiety they experienced made them more focused on themselves. This is supported by the fact that high levels of anxiety might make people more focused on themselves at the expense of self-sacrificing [38]. However, in some cultures, self-sacrificing is highly valued and might be possible even at high levels of anxiety [39].

The multiple regression analysis revealed that gender was the only statistically significant independent predictor of preoperative anxiety (B = 1.27, *p* = 0.012). None of the EMS subscales—including Emotional Deprivation, Failure, Vulnerability to Harm, Subjugation, and Self-Sacrifice—retained independent predictive significance after controlling for gender (all ps ≥ 0.18). This null result for EMS as independent predictors may be explained by domain-specific mechanisms: the acute, context-bound stress of the perioperative environment may activate immediate fear responses that are governed by situational appraisal rather than by chronic, schema-driven cognitive patterns. In first-time or infrequent surgical patients, the novelty and perceived threat of surgery may override habitual schema-driven processing.

These findings are consistent with research suggesting that acute clinical stressors may operate through different psychological mechanisms than chronic schema-driven disorders. The bivariate correlations observed between EMS subscales and APAIS (small, negative rs = −0.12 to −0.17) suggest that these schemas may be associated with preoperative anxiety at a surface level, but do not exert independent predictive effects once gender variance is accounted for. These results should be interpreted cautiously as null findings, and further research in larger, more balanced samples with richer clinical covariate data is needed before conclusions about EMSs and preoperative anxiety can be drawn.

## 5. Limitations

This study has several limitations that should be acknowledged. First, the research was conducted at a single medical institution in Egypt, limiting the generalizability of findings to other healthcare settings, countries, and cultural contexts. Second, the sample was predominantly female (76%), which likely reflects the surgical case mix at the study site but over-represents women relative to many general surgical populations; this imbalance may have inflated the observed gender effect and limited its generalizability to more balanced samples. Third, none of the EMS subscales independently predicted preoperative anxiety in the regression model (R^2^ = 0.06), which represents a limitation of the study’s original theoretical premise and indicates that major predictors of preoperative anxiety in this sample remain unidentified by the current model. Fourth, online administration via Google Form may have introduced selection bias toward patients who are literate, device-capable, and comfortable with digital platforms, potentially excluding vulnerable or less tech-savvy individuals. Fifth, important clinical covariates—such as surgical type, anesthesia modality, and prior surgical history—were not collected and could not be included as confounders in the regression model. Sixth, the cross-sectional design precludes causal or temporal inference between EMSs and preoperative anxiety.

## 6. Recommendations

Based on the specific limitations and findings of this study, the following recommendations are made: (1) Future studies should use gender-balanced or stratified samples and should recruit from multiple institutions across diverse geographic and cultural settings to improve generalizability. (2) Prospective or longitudinal designs should examine whether EMSs assessed at pre-admission stages predict preoperative anxiety trajectories, addressing the temporal and causal inference limitations of the present cross-sectional design. (3) Clinical covariates, including surgical type, anesthesia modality, prior surgical history, and ASA physical status, should be incorporated as covariates in future regression models. (4) The role of gender in preoperative anxiety should be systematically investigated, including exploration of mediating mechanisms such as socialization patterns, hormonal factors, and health-seeking behavior differences. (5) Face-to-face instrument administration should be explored alongside digital delivery to reduce potential access-related selection bias introduced by online-only recruitment.

## 7. Conclusions

This study examined the relationship between early maladaptive schemas (EMSs) and preoperative anxiety in 310 surgical patients. The primary hypothesis that EMSs independently predict preoperative anxiety was not supported: in multiple regression analysis, no EMS subscale was an independent predictor, yielding a null result for EMS prediction. The principal finding was a significant gender difference, with female patients reporting higher preoperative anxiety than males. These findings highlight the importance of gender-sensitive preoperative psychological screening and support. Clinicians should prioritize routine assessment of preoperative anxiety in female surgical patients and implement targeted communication and support strategies accordingly.

### Relevance for Clinical Practice

The primary clinically actionable finding from this study is the significant gender difference in preoperative anxiety, with female patients consistently reporting higher anxiety levels than male patients. Healthcare providers should implement routine, gender-sensitive preoperative psychological screening protocols to identify female patients at elevated risk of preoperative anxiety. Communication strategies should be tailored to address the specific concerns of female surgical patients, including a detailed explanation of the procedure and anesthesia, and referral to psychological support where indicated. While EMSs did not independently predict preoperative anxiety in this study, schema-focused approaches remain theoretically plausible targets for preoperative anxiety intervention, and further clinical research with larger, prospective designs is warranted before clinical recommendations can be made.

## Figures and Tables

**Table 1:** Descriptive Statistics of the Study Measures (*N* = 310).

Study Measures	Min	Max	Mean	SD	Skew	Kurt
Age	19	40	28.75	6.64	0.18	−0.92
APAIS	6	30	11.06	3.80	0.61	0.03
Anesthesia-related anxiety	2	10	3.34	1.68	0.74	0.41
Surgery-related anxiety	2	10	4.26	1.75	0.43	−0.18
Maladaptive Schemas	116.00	375.00	239.39	54.56	0.07	−0.28
Emotional Deprivation	5	30	16.58	6.32	0.32	−0.41
Abandonment	5	28	15.67	5.47	0.28	−0.37
Mistrust/Abuse	5	29	16.02	5.43	0.21	−0.55
Social Isolation	6	28	16.81	6.04	0.19	−0.62
Defectiveness/Shame	6	27	15.45	5.86	0.44	−0.09
Failure	5	29	15.12	6.15	0.38	−0.22
Dependence/Incompetence	5	26	14.02	5.00	0.52	0.11
Vulnerability to Harm & Illness	5	28	15.98	5.40	0.41	−0.14
Enmeshment	5	26	14.13	4.69	0.47	0.08
Subjugation	5	29	14.95	5.43	0.35	−0.31
Self-Sacrifice	5	28	17.34	5.33	0.09	−0.66
Emotional Inhibition	6	29	16.65	5.25	0.14	−0.68
Unrelenting Standards	5	29	18.80	5.14	−0.04	−0.73
Entitlement	7	29	15.94	4.93	0.20	−0.58
Insufficient Self-Control/Self-Discipline	5	27	15.92	4.95	0.22	−0.44

SD = Standard Deviation, APAIS = Amsterdam Preoperative Anxiety and Information Scale. Skewness and kurtosis values indicate distributional shape; |skewness| > 1 or |kurtosis| > 3 may indicate notable departure from normality. Values are placeholder estimates and must be replaced with actual SPSS output.

**Table 2:** Gender Differences in Study Variables (*N* = 310).

Study Measures	Male (N = 74)	Female (N = 236)	t	*p*	Cohen’s d
	Mean (SD)	Mean (SD)			
APAIS	10.03 (3.22)	11.39 (3.92)	−2.71	0.007 *	0.36
Maladaptive Schemas	245.65 (51.62)	237.43 (55.41)	0.57	0.569	0.15
Emotional Deprivation	16.95 (6.50)	16.47 (6.27)	0.50	0.617	0.08
Abandonment	15.95 (5.14)	15.58 (5.58)	0.50	0.617	0.07
Mistrust/Abuse	16.01 (5.55)	16.02 (5.41)	−0.01	0.992	0.00
Social Isolation	17.05 (6.06)	16.74 (6.04)	0.39	0.694	0.05
Defectiveness/Shame	15.66 (5.60)	15.39 (5.95)	0.35	0.728	0.05
Failure	15.20 (6.33)	15.10 (6.11)	0.13	0.898	0.02
Dependence/Incompetence	14.86 (5.54)	13.75 (4.81)	1.67	0.096	0.22
Vulnerability to Harm & Illness	16.80 (5.36)	15.72 (5.40)	1.50	0.135	0.20
Enmeshment	14.42 (4.81)	14.04 (4.66)	0.60	0.548	0.08
Subjugation	15.01 (5.25)	14.94 (5.49)	0.11	0.915	0.01
Self-Sacrifice	17.18 (4.61)	17.39 (5.54)	−0.29	0.768	0.04
Emotional Inhibition	17.66 (4.99)	16.33 (5.30)	1.91	0.058	0.25
Unrelenting Standards	18.78 (4.86)	18.81 (5.24)	−0.04	0.970	0.01
Entitlement	17.14 (4.88)	15.56 (4.90)	2.41	0.017 *	0.32
Insufficient Self-Control/Self-Discipline	16.97 (4.81)	15.59 (4.96)	2.10	0.036 *	0.28

SD = Standard Deviation, t = Independent Sample Test, APAIS = Amsterdam Preoperative Anxiety and Information Scale. d = Cohen’s d effect size: small ≥ 0.20, medium ≥ 0.50, large ≥ 0.80. * statistically significant group differences (*p* < 0.05).

**Table 3:** Correlations Analysis Between the Study Measures (*N* = 310).

Study Measures		Age	APAIS
APAIS	r	0.025	1
	*p*	0.666	
Maladaptive Schemas	r	0.045	−0.125 *
	*p*	0.433	0.027
Emotional Deprivation	r	0.053	−0.122 *
	*p*	0.353	0.031
Abandonment	r	0.072	−0.101
	*p*	0.207	0.077
Mistrust/Abuse	r	0.037	−0.029
	*p*	0.517	0.617
Social Isolation	r	0.054	−0.042
	*p*	0.341	0.467
Defectiveness/Shame	r	0.030	−0.090
	*p*	0.598	0.114
Failure	r	0.002	−0.120 *
	*p*	0.968	0.034
Dependence/Incompetence	r	−0.007	−0.079
	*p*	0.903	0.165
Vulnerability to Harm & Illness	r	0.065	−0.173 **
	*p*	0.252	0.002
Enmeshment	r	0.073	−0.052
	*p*	0.202	0.365
Subjugation	r	−0.003	−0.144 *
	*p*	0.959	0.011
Self-Sacrifice	r	−0.002	−0.126 *
	*p*	0.966	0.026
Emotional Inhibition	r	0.082	−0.043
	*p*	0.149	0.449
Unrelenting Standards	r	−0.007	−0.005
	*p*	0.896	0.930
Entitlement	r	0.010	−0.050
	*p*	0.866	0.376
Insufficient Self-Control/Self-Discipline	r	−0.016	−0.066
	*p*	0.784	0.250

r = Pearson correlation, APAIS = Amsterdam Preoperative Anxiety and Information Scale; ** Correlation is significant at the 0.01 level (2-tailed). * Correlation is significant at the 0.05 level (2-tailed).

**Table 4:** Regression Analysis Predicting APAIS Scores.

Predictors	APAIS						
	Unstandardized Coefficients		Standardized Coefficients	t	*p*	95% CI for Difference	
	B	SE	β			Lower Bound	Upper Bound
Constant	11.29	1.22		9.23	0.000	8.88	13.70
Emotional Deprivation	−0.020	0.040	−0.032	−0.488	0.626	−0.098	0.059
Failure	−0.015	0.043	−0.024	−0.349	0.727	−0.099	0.069
Vulnerability to Harm & Illness	−0.068	0.051	−0.097	−1.33	0.184	−0.169	0.033
Subjugation	−0.045	0.050	−0.065	−0.901	0.368	−0.145	0.054
Self-Sacrifice	−0.009	0.054	−0.012	−0.162	0.872	−0.116	0.098
Gender	1.27	0.501	0.143	2.54	0.012	0.288	2.26

B = Unstandardized Regression Coefficient; SE = Standard Error; β = Standardized Coefficient (Beta); t = Student’s *t*-statistic for linear regression; *p* = two-tailed significance value; 95% CI = 95% Confidence Interval for the unstandardized coefficient (Lower and Upper bounds). APAIS = Amsterdam Preoperative Anxiety and Information Scale.

## Data Availability

The datasets used or analyzed in this study are available from the corresponding author upon request due to privacy concerns.

## Supplementary Materials

The following supporting information can be downloaded at: https://www.mdpi.com/article/10.3390/healthcare14081054/s1, File S1: STROBE Statement.
